# Targeted antitumor prodrug therapy using CNGRC-yCD fusion protein in combination with 5-fluorocytosine

**DOI:** 10.1186/s12929-016-0227-6

**Published:** 2016-01-22

**Authors:** Jia-Je Li, Shun-Fu Chang, I-Iu Liau, Pei-Chia Chan, Ren-Shyan Liu, Sang-Hue Yen, Hsin-Ell Wang, Cheng Allen Chang

**Affiliations:** Department of Biomedical Imaging and Radiological Sciences, National Yang-Ming University, No. 155, Sec. 2, Linong St., Beitou District, Taipei, 112 Taiwan, ROC; Program in Molecular Medicine, National Yang-Ming University and Academia Sinica, Taipei, 112 Taiwan, ROC; Department of Medical Research and Development, Chang Gung Memorial Hospital-Chiayi Branch, Chiayi, 613 Taiwan, ROC; Department of Nuclear Medicine and National PET/Cyclotron Center, Taipei Veterans General Hospital, Taipei, 112 Taiwan, ROC; Department of Oncology Medicine, Taipei Veterans General Hospital, Taipei, 112 Taiwan, ROC; Biophotonics & Molecular Imaging Research Center, National Yang-Ming University, Taipei, 112 Taiwan, ROC; Biomedical Engineering Research and Development Center, National Yang-Ming University, Taipei, 112 Taiwan, ROC

**Keywords:** Targeted cancer therapy, Aminopeptidase N, Asparagine-glycine-arginine motif, Cytosine deaminase, 5-fluorocytosine, 5-fluorouracil

## Abstract

**Background:**

The enzyme-prodrug system is considered a promising tool for tumor treatment when conjugated with a targeting molecule. The asparagine-glycine-arginine (NGR) motif is a developing and interesting targeting peptide that could specifically bind to aminopeptidase N (APN), which is an NGR receptor expressed on the cell membrane of angiogenic endothelial cells and a number of tumor cells within the tumor tissues. The objective of this study was to develop a novel targeted enzyme-prodrug system using 5-fluorocytosine (5-FC) and an NGR-containing peptide fused with yeast cytosine deaminase (yCD), i.e. CNGRC-yCD fusion protein, to target APN-expressing cells within the tumor tissues and to convert 5-FC into 5-fluorouracil (5-FU) to kill tumors.

**Results:**

Both yCD and CNGRC-yCD proteins were cloned into the pET28a vector and expressed by an *Escherichia coli* host. Both yCD and CNGRC-yCD proteins were purified and the yields were approximately 20 mg/L with over 95 % purity. The binding assay demonstrated that the CNGRC-yCD fusion protein had specific binding affinity toward purified APN recombinant protein and high-APN-expressing cells, including human endothelial cells (HUVECs) and various types of human tumor cell lines, but not low-APN-expressing tumor cell lines. Moreover, the enzyme activity and cell viability assay showed that the CNGRC-yCD fusion protein could effectively convert 5-FC into 5-FU and resulted in significant cell death in both high-APN-expressing tumor cells and HUVECs.

**Conclusions:**

This study successfully constructs a new targeting enzyme-prodrug system, CNGRC-yCD fusion protein/5-FC. Systematic experiments demonstrated that the CNGRC-yCD protein retained both the APN-binding affinity of NGR and the enzyme activity of yCD to convert 5-FC into 5-FU. The combined treatment of the CNGRC-yCD protein with 5-FC resulted in the significantly increased cell death of high-APN-expressing cells as compared to that of low-APN-expressing cells.

## Background

Angiogenesis is an indispensable process for tumor growth and metastasis [1, 2]. Anti-angiogenic therapy targeting the angiogenic endothelial cells within tumors has been an important and continuously developing strategy against cancer [3–5]. The asparagine-glycine-arginine (NGR) motif found by phage display libraries since the 1990s is a highly specific tumor-homing peptide that targets the aminopeptidase N (APN) on the surface of neo-angiogenic but not normal endothelial cells [6–11]. Subsequent studies have demonstrated that the NGR motif could be a potent delivery vehicle to carry cytotoxic drugs and probes to the tumor tissues for tumor-targeting treatment and diagnosis, respectively. For example, tumor necrosis factor-α (TNF-α), interferon-γ, tissue factor (TF) and doxorubicin have been linked with NGR-containing peptides and have exerted effective antitumor efficacies on APN-expressing cells [7, 12–14]. In addition, the cyanine dye Cy5.5 conjugated with an NGR-containing peptide showed in vivo affinity to APN-expressing cells and may serve as a promising molecular imaging probe [15, 16]. Besides being expressed in the angiogenic endothelial cells, APN recently has been found in multiple types of tumor cells, a fact that plays an important role in modulating tumor metastasis and survival [16].

The enzyme-prodrug system that produces active drugs from safer prodrugs at the tumor site is an attractive strategy for antitumor therapy [17–24]. The combined therapy employing cytosine deaminase (CD) and the nucleoside analog 5-fluorocytosine (5-FC) is an effective approach offered by the enzyme-prodrug system. CD was endogenously expressed in yeast and bacteria but not in mammalian cells and could efficiently convert the less-toxic 5-FC into the more-cytotoxic pyrimidine analog 5-fluorouracil (5-FU), which would lead to the inhibition of nucleotide and protein synthesis in tumor cells [17–20]. Moreover, it has been proposed that the transformation efficiency of 5-FC into 5-FU by yeast CD, i.e. yCD, is much better than that by bacteria [21, 22], making the yCD/5-FC system a better choice for antitumor therapy. In the past, significant in vitro and in vivo studies on the inhibition of tumor growth and cell death have been reported after the mammalian cell transfection of CD gene therapy and administration of 5-FC [19, 23, 24]. On the other hand, our studies and those of others have demonstrated that the CD fusion protein conjugates of epidermal growth factor (EGF) [20] or endostatin [25] maintain high CD enzyme activity to convert 5-FC into 5-FU and have showed effective targeted antitumor potency.

In this paper, we report the preparation and characterization of a novel tumor-targeted enzyme-prodrug system that includes 5-FC and the fusion protein CNGRC-yCD. The purified CNGRC-yCD protein expressed by an *Escherichia coli* host retained effective NGR-APN binding affinity and high CD enzyme activity to convert 5-FC into 5-FU. The combined treatment of CNGRC-yCD fusion protein and 5-FC prodrug resulted in the significant cell death of types of high-APN-expressing human tumor cell lines and endothelial cells (HUVECs), but it did not result in the cell death of low-APN-expressing human tumor cell lines. This suggests that the newly developed CNGRC-yCD fusion protein in combination with 5-FC has potential as an APN-targeting antitumor enzyme-prodrug system.

## Methods

### Materials

MDA-MB468 (human breast adenocarcinoma) and HT-29 (human colorectal adenocarcinoma) cell lines were purchased from American Type Culture Collection. MDA-MB231 (human breast adenocarcinoma), MCF7 (human breast adenocarcinoma), A431 (human epidermoid carcinoma), A375 (human malignant melanoma), A549 (human lung carcinoma), HT-1080 (human fibrosarcoma) and HUVECs (human umbilical vein endothelial cells) cell lines were purchased from Bioresource Collection and Research Center in Taiwan. The ES2 cell line (human ovarian carcinoma) was a kind gift from Dr. Chi-Mu Chuang (Department of Obstetrics and Gynecology, Taipei Veterans General Hospital). Cell culture materials were obtained from Thermo Scientific Inc. (HyClone Laboratories, Inc., Logan, UT, USA). An EGM™-2 Endothelial Cell Growth Medium-2 Bullet kit was purchased from Lonza, Inc. (Walkersville, MD, USA). A nitrilotriacetic acid (NTA) column (HisTrap FF crude) and size exclusion column (HiPrep 26/60 Sephacryl S-100 High Resolution) for purification were purchased from GE Healthcare Corporation (Uppsala, Sweden). Coomassie brilliant blue was purchased from Sigma-Aldrich Chemical Corporation (St. Louis, MO, USA). A Bio-Rad protein assay kit (#500-0002) was acquired from Bio-Rad Laboratories (Hercules, CA, USA). Complete mini-ethylenediaminetetraacetic acid (EDTA)–free protease inhibitor cocktail tablets were purchased from Roche Corporation (Indianapolis, IN, USA). An anti-His_6_ tag horseradish peroxidase (HRP) labeled mouse monoclonal antibody was purchased from R&D Systems (Minneapolis, MN, USA). Anti-human aminopeptidase N antibody (clone WM15) was purchased from BD Biosciences, Inc. (San Jose, USA). Alexa 488 conjugated goat anti-mouse immunoglobulin G (IgG) secondary antibody was obtained from Life Technologies, Inc. (Eugene, OR, USA). Human aminopeptidase N recombinant protein was obtained from Abnova, Inc. (Taipei, Taiwan). All other chemicals were purchased from Merck & Co., Inc. (Whitehouse Station, NJ, USA).

### Cloning of DNA in the expression vector

The DNA sequence encoding yCD and CNGRC-yCD proteins was amplified by polymerase chain reaction (PCR) using a complementary DNA (cDNA) library that was obtained from yeast as a template. The sense and antisense primers used for the amplification of yCD were 5′-TATACCATGGTGGTCACAGGAGGCATGG-3′ and 5′-TTACTCGAGCTCCCCAATG TCCTCAAAC-3′, which introduced *Xho*I and *Nco*I restriction enzyme sites, respectively. To construct the CNGRC-yCD fusion gene, the two genes were linked via a two-amino-acid residue linker sequence GG. 5′-TACCATGGGTTGCAACGGTCGTTGTGGTGGTGTCACAGGAGGCATGG-3′ and 5′-TTACTCGAGCTCCCCAATGTCCTCAAAC-3′ were used as sense and antisense primers that introduced *Xho*I and *Nco*I restriction enzyme sites to clone CNGRC-yCD. The resulting PCR products were cut with *Xho*I and *Nco*I and ligated into the protein expression vector, pET28a, which was cut with the same enzymes. The C-terminus of the pET28a vector has a hexa-histidine (His_6_) tag for convenient protein recognition and purification.

### Expression and purification of yCD and CNGRC-yCD proteins

The pET28a-CNGRC-yCD and pET28a-yCD plasmids were transformed into competent BL21 (DE3) *Escherichia coli*. The yCD and CNGRC-yCD genes were expressed by a T7-RNA polymerase-controlled bacterial system using BL21 (DE3) *Escherichia coli* at 16 °C with Luria-Bertani (LB) broth containing 0.5 mM znic acetate and 1 mM isopropyl β-D-1-thiogalactopyranoside (IPTG) for induction at an OD_600_ nm of 0.5–0.6. Cells were harvested by centrifugation for 10 min at 4 °C. The pellet was resuspended in 100 mL resuspension buffer (20 mM Tris, 500 mM NaCl, 20 mM imidazole, 0.5 mM phenylmethylsulfonyl fluoride (PMSF), 250 μg/mL lysozyme, 10 μg/mL deoxyribonuclease (DNase) I, 5 mM MgCl_2_, pH 8.0) and incubated for 60 min at 25 °C. The suspension was sheared by a French Press dispersing apparatus with 30 kPSI (pounds per square inch). The mixture was centrifuged at 4 °C for 30 min. The supernatant containing the soluble recombinant proteins was harvested and pumped onto a Ni-NTA column using the ӒKTA FPLC P-920 purification system. Proteins were eluted in a two-step linear gradient of imidazole (first step at concentrations of 50 to 100 mM and second step at concentrations of 100 to 500 mM). The peak fractions of the 200 to 250 mM imidazole eluates were pooled and subjected to further purification using a gel filtration column. The proteins were characterized on 10 % sodium dodecyl sulfate polyacrylamide gel electrophoresis (SDS–PAGE) gels by staining with Coomassie brilliant blue. Protein concentrations were determined by Bio-Rad protein assay kits according to the manufacturer’s instructions.

### Cell culture

All cells except the last two were grown at 37 °C in a 5 % CO_2_ incubator. The A375 and A431 cells were grown in DMEM containing 10 % fetal bovine serum (FBS). The MCF-7 and HT-1080 cells were grown in MEM containing 10 % FBS and 1 mM MEM nonessential amino acid. The ES2 cells were grown in RPMI 1640 containing 10 % FBS. The HT-29 cells were grown in McCoy’s 5A medium containing 10 % FBS. The A549 cells were grown in F-12 K medium containing 10 % FBS. The HUVECs were grown in Medium 199 containing 20 % FBS and 10 % EGM™-2 Endothelial Cell Growth Medium-2 [26]. The MDA-MB231 and MDA-MB468 cells were grown in L-15 medium containing 10 % FBS at 37 °C in a 0 % CO_2_ incubator. For preparing the cells used in cell binding assay and MTT assay, cancer cells and HUVECs were grown in the same culture conditions as described above.

### Evaluation of yCD and CNGRC-yCD enzyme kinetics on 5-FC/5-FU transformation

The enzymatic activities of yCD and CNGRC-yCD proteins were determined by measuring the production rates of 5-FU in the presence of various amounts of 5-FC. 118 nM of yCD or CNGRC-yCD protein was mixed with increasing concentrations of pre-warmed 5-FC (0.181, 0.363, 0.725, 1.5, and 3.0 mM) in phosphate-buffered saline solutions (PBS, 0.01 M phosphate, 0.138 M NaCl, 2.7 mM KCl, pH 7.4) to initiate the conversion of 5-FC to 5-FU at 37 °C for 0.5, 1.0, 2.0 and 3.0 min. Then, the reactions were quenched by adding 0.2 N HCl solutions. One mL of each reaction solution was sampled and the concentrations of 5-FC and 5-FU were determined using a DU800 ultraviolet–visible (UV/VIS) spectrophotometer (Beckman Coulter). The absorbance values at wavelengths of 255 nm and 290 nm were used to calculate the concentrations of 5-FU and 5-FC, using a formula previously deduced as follows:

$$ 5\hbox{-} \mathrm{F}\mathrm{C}\ \left[\mathrm{mmol}/\mathrm{L}\right] = 0.119 \times {\mathrm{A}}_{290} - 0.025 \times {\mathrm{A}}_{255} $$

$$ 5\hbox{-} \mathrm{F}\mathrm{U}\ \left[\mathrm{mmol}/\mathrm{L}\right] = 0.185 \times {\mathrm{A}}_{255} - 0.049 \times {\mathrm{A}}_{290} $$

The rates of 5-FU production under various conditions by either yCD or CNGRC-yCD protein were used to calculate the *V*_*max*_, K_m_ and *k*_*cat*_ values by using GraphPad Prism (GraphPad Software, San Diego, CA).

### In vitro binding of the yCD and CNGRC-yCD proteins to immobilized APN

The purified recombinant human APN protein was diluted in PBS (0.5 μg/mL) and immobilized on a 96-well enzyme-linked immunosorbent assay (ELISA) plate by incubation at 4 °C overnight. The wells were washed three times with PBST (phosphate buffer saline with 0.05 % Tween 20 solution), followed by blocking with the addition of 300 μL 5 % bovine serum albumin (BSA) in PBS at ambient temperature for 1 h. Then, the plate was rewashed three times with PBST before the addition of the yCD or CNGRC-yCD protein solutions at various concentrations (i.e. 4.0, 2.0, 1.0, 0.5, 0.25, 0.125 and 0.0625 μM). The ligands (yCD or CNGRC-yCD) and APN proteins were incubated at ambient temperature for 2 h, and the plate was then washed twice with PBST, followed by the addition of 100 μL anti-His_6_-HRP monoclonal antibody diluted in 1 % BSA (1:1000). After incubation at ambient temperature for 1 h, the plate was washed with PBST twice, followed by the addition of 100 μL of the HRP substrate (i.e. 3,3′,5,5′-tetramethylbenzidine, TMB) to each well. The reaction was terminated after 15 min at ambient temperature by the addition of 50 μL of the stop solution (2 N H_2_SO_4_) to each well. The optical density (OD_450_) in each well was determined using an ELISA plate reader. The binding affinity in μM was computed using GraphPad Prism (GraphPad Software, San Diego, CA) by nonlinear regression analysis.

### Examination of the APN expression level in various cell lines by flow cytometry

The levels of APN expression in various human tumor cell lines and human umbilical vein endothelial cells (HUVECs) were analyzed by FACScan flow cytometry (Becton-Dickinson). Tumor cells were grown to 90 % confluent and HUVECs were grown to 50 % or 90 % confluent. Then, the cells were harvested (~1 x 10^6^), washed, and probed with anti-APN antibody (WM15) on ice for 1 h. After the unbound first antibody was removed by washing with ice-cold PBS three times, the surface-bound antibody was visualized by probing the cells with goat anti-mouse IgG secondary antibody conjugated with Alexa488 for 1 h on ice and was analyzed using a FACScan flow cytometer (Becton-Dickinson). Three repeats were done for each cell line.

### In vitro cell binding assay of yCD and CNGRC-yCD proteins in cell lines expressing different levels of APN

The cells expressing different levels of APN were seeded in a 96-well plate with a density of 20,000 cells/well. After 12 h of incubation, the cells were fixed by pre-cooling para-formaldehyde for 15 min and blocked with fetal bovine serum for 1 h. Then, the plate was washed with PBST before the addition of His_6_-tagged yCD or CNGRC-yCD. To measure the dissociation constant (K_d_), the protein solutions at various concentrations (i.e. 2.0, 1.0, 0.5, 0.25, 0.125 and 0.0625 μM) were added to the HT-1080 or HT-29 cells. To test the relative binding capacities of various cells, 100 μL of 2.0 μM solutions of yCD or CNGRC-yCD were added to each cell described previously in the materials section. The recombinant proteins and cells were incubated at ambient temperature for 1 h, and the plate was then washed twice with PBST, followed by the addition of mouse anti-His_6_-HRP monoclonal antibody. After incubation at ambient temperature for 1 h, the plate was washed with PBST twice, followed by the addition of TMB to each well. The reaction was terminated after 20 min by the addition of the stop solution (2 N H_2_SO_4_ solutions) to each well. The optical density (OD_450_) in each well was determined using an ELISA plate reader. The binding affinity in μM was computed using GraphPad Prism (GraphPad Software, San Diego, CA) by nonlinear regression analysis.

### MTT assay of the cell viability after yCD or CNGRC-yCD protein/5-FC treatment

The cells were seeded in a 96-well plate and treated with 100 μL of 2 μM solutions of yCD or CNGRC-yCD protein. After 1 h incubation, the unbound proteins were removed and the cells were washed with PBS three times and incubated with various concentrations of 5-FC (0.1, 1, 10, 100, and 1000 μM). Cells of the control groups were only incubated with different concentrations of 5-FU or 5-FC (0.1, 1, 10, 100 and 1000 μM) without yCD or CNGRC-yCD protein treatment. Cells were subjected to the MTT (3-(4,5-dimethylthiazol-2-yl)-2,5-diphenyltetrazolium bromide, 0.5 mg/mL in culture medium) assay 3 days after the addition of the indicated proteins and 5-FC or 5-FU. After 1 h of incubation, the MTT solution was removed and the cells were washed with PBS, followed by the addition of the stop solution (dimethyl sulfoxide, DMSO). After 15 min incubation at ambient temperature, the optical density (OD_570_) was measured with DMSO alone as a blank.

### Statistical analysis

Results were expressed as mean ± standard deviation of the mean (SD). Statistical analysis was performed by using an independent Student *t*-test for the two groups of data. A *P* value less than 0.05 was considered significant.

## Results

### Design, expression, and purification of CNGRC-yCD fusion protein

**Fig. 1:**
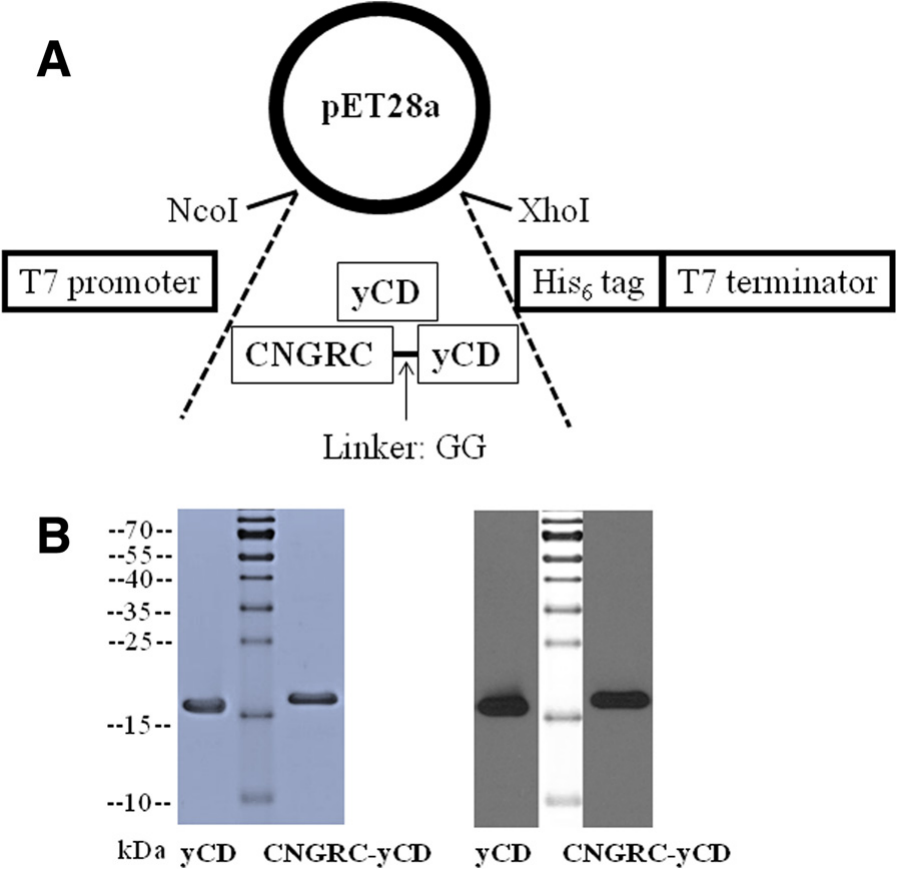
Schematic diagram of gene construction and identification of fusion proteins. **a** The genes encoding CNGRC-yCD and yCD were cloned into the pET28a expression vector using a PCR cloning strategy, and digested with the restriction enzymes, *Nco* I and *Xho* I. **b** The respective purified yCD and CNGRC-yCD fusion proteins were identified by Coomassie brilliant blue stained gels (left panel) and Western blot using His_6_-tag-specific antibody (right panel). The experiments were completed at least three times with similar results

Previous studies have shown that the affinity of the cyclic CNGRC formed by the disulfide bond formation of the two terminal cysteine groups on APN-expressing cells is greater than that of linear NGR [10]. Therefore, the cyclic CNGRC was chosen to be fused with yCD and cloned into the pET28a expression vector using a PCR cloning strategy. The dipeptide GG was used as the linker between CNGRC and yCD (Fig. 1a). The control was yCD protein alone. Both yCD and CNGRC-yCD proteins were expressed by an *Escherichia coli* host. The C-terminus of both proteins consisted of a hexa-histidine (His_6_) sequence for convenient protein purification and detection. The yields of yCD and CNGRC-yCD proteins in soluble forms were ~20 mg/L with over 95 % purity. The purified proteins were identified by Coomassie brilliant blue stained gels (left panel, Fig. 1b) and Western blot using His_6_-tag-specific antibody (right panel, Fig. 1b).

### CNGRC-yCD fusion protein retains binding affinity to APN protein

**Fig. 2:**
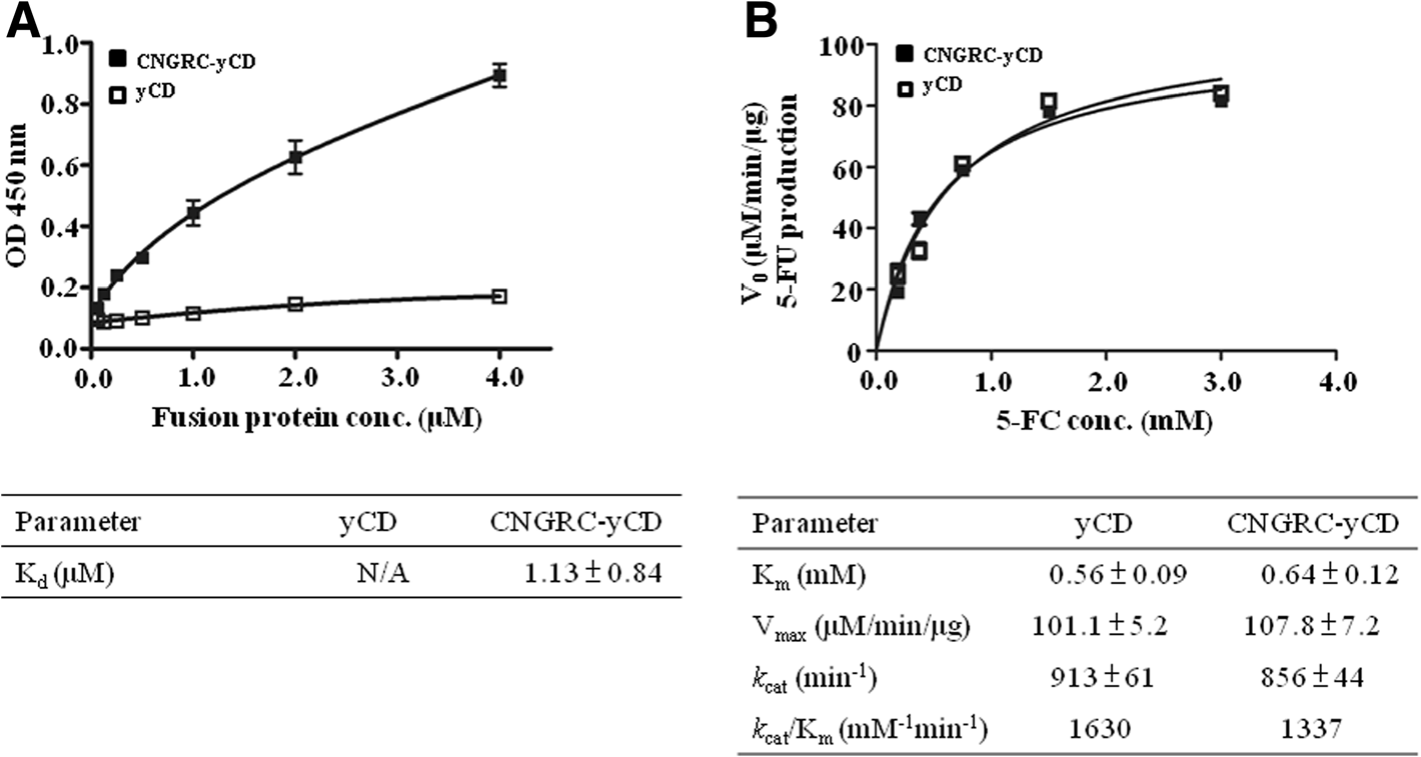
Enzyme activities of yCD and CNGRC-yCD proteins to transform 5-FC to 5-FU and the binding affinity of both proteins to the recombinant APN. **a** The recombinant APN protein was coated on a 96-well plate and then an increasing dose of protein solutions (0.0625, 0.125, 0.25, 0.5, 1.0, 2.0 and 4.0 μM) was added into the wells. The binding affinities of both yCD and CNGRC-yCD proteins to APN were determined by ELISA assay using horseradish peroxidase (HRP)–tagged anti-His_6_ antibody. **b** Increasing concentrations of 5-FC solutions (0.181, 0.363, 0.725, 1.5 and 3.0 mM) were added into a solution containing 50 nM of yCD or CNGRC-yCD fusion protein. The concentrations of 5-FC and 5-FU were determined by measuring the UV absorbance (λ_255_ and λ_290_) at 0.5, 1.0, 2.0 and 3.0 min after mixing 5-FC with the proteins. At least three repeats were performed with similar results

The binding affinities of both yCD and CNGRC-yCD proteins to the APN protein were determined by ELISA assay using horseradish peroxidase (HRP)-tagged anti-His_6_ antibody. The APN protein was coated on a 96-well plate and the protein solutions at various concentrations (i.e. 4.0, 2.0, 1.0, 0.5, 0.25, 0.125 and 0.0625 μM) were added into the wells. The results showed that the CNGRC-yCD protein has an APN binding affinity with a K_d_ value of 1.13 ± 0.84 μM (Fig. 2a). In contrast, the yCD protein showed only insignificant APN binding affinity as expected (Fig. 2a).

### CNGRC-yCD fusion protein retains CD enzyme activity to convert 5-FC into 5-FU

The enzyme activities of yCD and CNGRC-yCD proteins can be confirmed by the transformation rates. Figure 2b shows that the 5-FC to 5-FU transformation rates were 5-FC dose dependent and the efficiency by yCD and CNGRC-yCD proteins was similar. The respective kinetic parameters for the catalysis of 5-FC to 5-FU by yCD and CNGRC-yCD determined are *V*_*max*_, 101.1 ± 5.2 and 107.8 ± 7.2 μm/min/μg; K_m_ values, 0.56 ± 0.09 and 0.64 ± 0.12 mM; *k*_*cat*_ values, 913 ± 61 and 856 ± 44 min^−1^; *k*_*cat*_/K_m_ values, 1630 and 1337 mM^−1^min^−1^.

### CNGRC-yCD fusion protein retains binding affinity and selectivity on APN-expressing cells

**Fig. 3:**
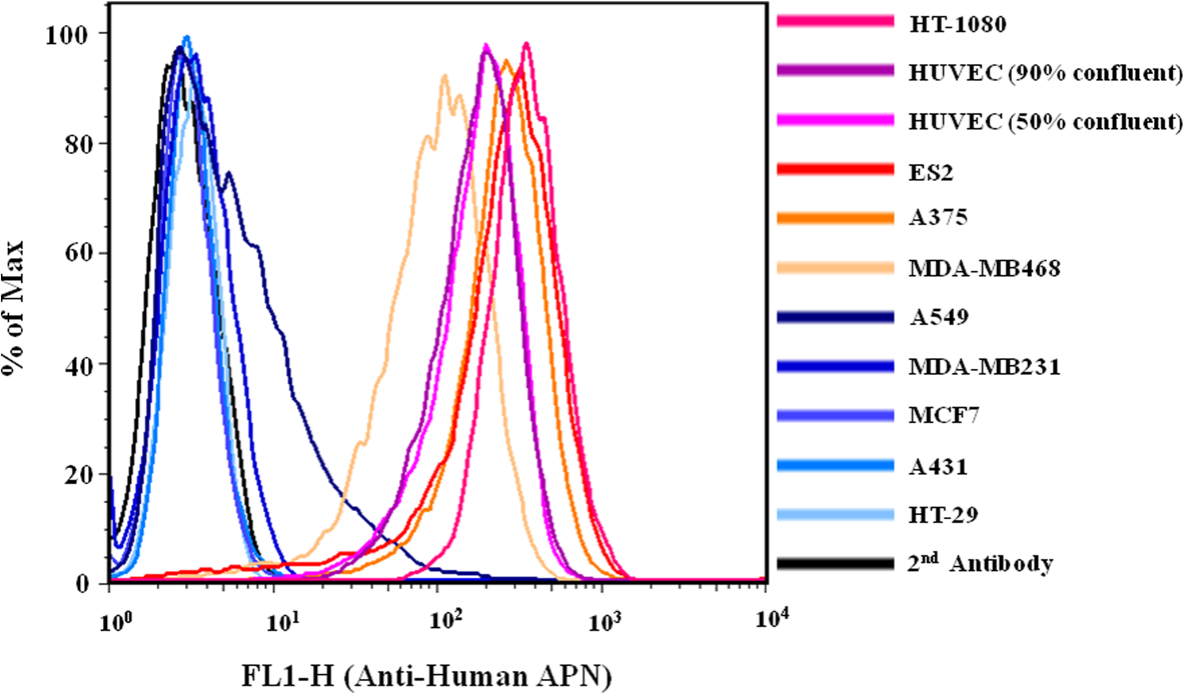
APN expression levels in various types of cell lines. Tumor cells were grown to 90 % confluent and HUVECs was grown to 50 % and 90 % confluent. Cells were probed with anti-APN antibody (WM15) and then with secondary antibody conjugated with Alexa488. The levels of APN expression for all cell lines were analyzed by flow cytometry. At least three repeats were performed with similar results

**Fig. 4:**
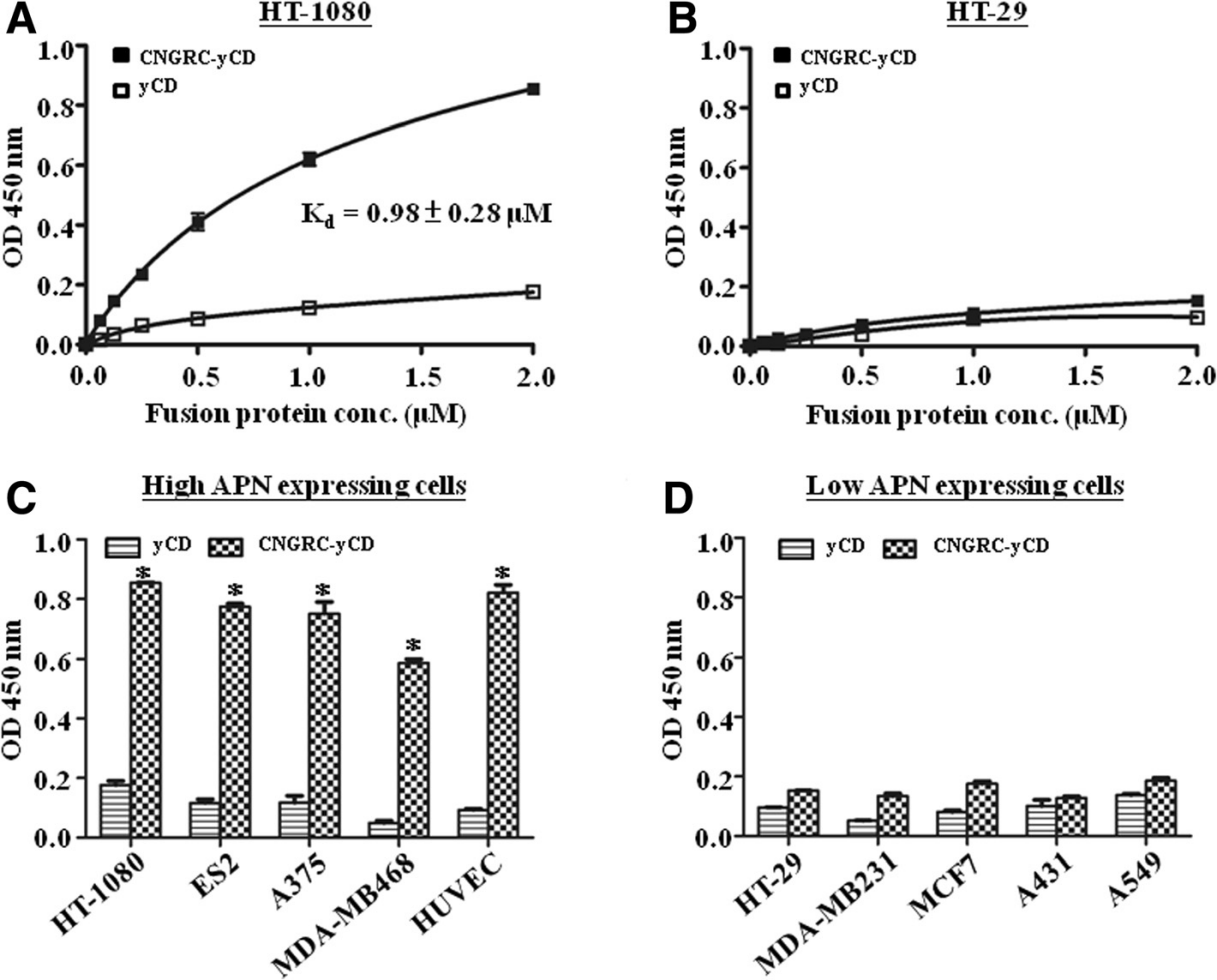
CNGRC-yCD fusion protein has high binding affinity to APN-expressing cells. **a**-**b**. HT-1080 **a**, and HT-29 **b**. cells were cultured on a 96-well plate. CNGRC-yCD or yCD protein (0.0625, 0.125, 0.25, 0.5, 1.0 and 2.0 μM) was added into the wells to determine its specific binding affinity by ELISA assay using horseradish peroxidase (HRP)–tagged anti-His_6_ antibody. **c**-**d**. High- **c**. and low- **d**. APN-expressing cells were cultured on a 96-well plate. 2.0 μM of yCD or CNGRC-yCD protein was added into the wells and the binding capacities of both proteins were determined by ELISA assay using horseradish peroxidase (HRP)–tagged anti-His_6_ antibody. At least three repeats were performed with similar results (*, *t* test, *P* < 0.05 relative to the yCD group)

The levels of APN expressions in various types of cancer cell lines and HUVECs were examined. The results showed that the levels of APN expressions are high in HT-1080, ES2, A375 and MDA-MB468 cancer cells and HUVECs but low in HT-29, A549, MDA-MB231, MCF7 and A431 cancer cells (Fig. 3). The HT-1080, the highest-APN-expressing cell line, and the HT-29, the lowest-APN-expressing cell line, were selected as representative cell lines. The dissociation constants (K_d_) of yCD and CNGRC-yCD proteins to these two cell lines were determined using ELISA assay with horseradish peroxidase (HRP)–tagged anti-His_6_ antibody. The CNGRC-yCD protein exhibited a remarkable binding affinity to the HT-1080 cells (K_d_ 0.98 ± 0.28 μM, Fig. 4a), which was similar to that with the APN protein (K_d_ 1.13 ± 0.84 μM, Fig. 2a), but it showed no specific binding to the HT-29 cells (Fig. 4b). The yCD protein, as expected, displayed only insignificant binding affinity to both cell lines (Fig. 4a and b). Moreover, the specific binding of yCD and CNGRC-yCD proteins to the high-APN-expressing (HT-1080, ES2, A375, MDA-MB468 and HUVECs) and low-APN-expressing (HT-29, MDA-MB231, MCF7, A431 and A549) cell lines was determined at a saturation concentration (2.0 μM) of yCD or CNGRC-yCD proteins. The results indicated that CNGRC-yCD protein exhibited specific binding to the cells with high APN-expression, but not to those with low APN-expression (Fig. 4c and d). Again, the yCD protein showed no specific binding to any of the types of tumor and endothelial cells chosen for this study (Fig. 4c and d).

### Pre-incubation with CNGRC-yCD fusion protein and then treatment with 5-FC significantly decreases the viability of APN-expressing cells

**Fig. 5:**
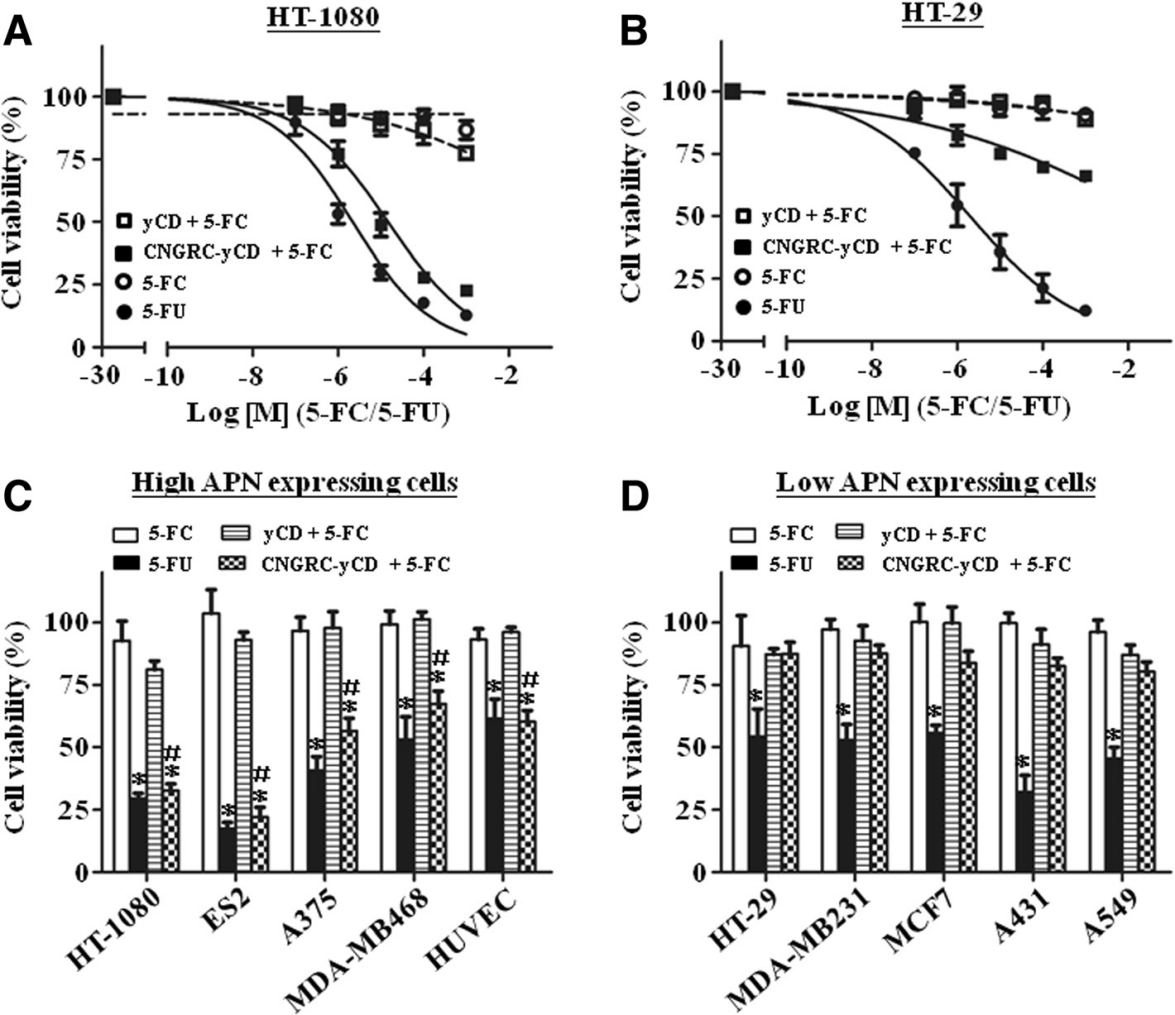
CNGRC-yCD/5-FC combination treatment significantly reduces cell viability of high-APN-expressing cells. **a**-**b**. HT-1080 **a**. and HT-29 cells **b**. were treated with 2.0 μM of proteins and with an increasing dose of 5-FC (0.1, 1.0, 10, 100 and 1000 μM). Then, the cell viability was determined by MTT assay. **c**-**d**. High-**c** and low-**d** APN-expressing cells were treated with 2.0 μM of protein and 60 μM of 5-FC and then the cell viability was determined by MTT assay. Treatments with 5-FC and 5-FU alone were used as negative and positive controls, respectively. At least three repeats were performed with similar results (*, *t* test, *P* < 0.05 relative to the 5-FC treatment group; #, *t* test, *P* < 0.05 relative to the yCD/5-FC treatment group)

To investigate the specificity and sensitivity of the CNGRC-yCD/5-FC enzyme-prodrug system for potential targeted tumor therapy, the viability of HT-1080 and HT-29 cells after a sequential treatment with yCD or CNGRC-yCD protein and 5-FC was determined by MTT assay. In the CNGRC-yCD/5-FC treatment group, the viability of HT-1080 cells decreased dramatically in response to an increasing dose of 5-FC (IC_50_ 14.8 ± 0.4 μM, Fig. 5a), but not for HT-29 cells (IC_50_ 39430.4 ± 347.2 μM, Fig. 5b). In the yCD/5-FC treatment group, the viability of both cell lines remained high despite the increasing dose of 5-FC, similar to that of 5-FC treatment alone. Throughout this study, 5-FU treatment was used as a positive control, and it did induce a significant cell death in both cell lines. Further treatment of all high- and low-APN-expressing cells with about 4 times the IC_50_ dose of 5-FC (60 μM) was performed after pre-incubation of 2.0 μM of yCD or CNGRC-yCD proteins. The results showed that in the CNGRC-yCD/5-FC treatment group, the viabilities of high-APN-expressing tumor cells and endothelial cells were significantly decreased as compared to the yCD/5-FC and 5-FC treatment group. Among the five high-APN-expressing cell lines, HT-1080 and ES2 seemed more sensitive to 5-FU compared with A375, MDA-MB468 and HUVECs. The decreased viability level of each cell line seemed to correlate to that of 5-FU treatment alone (Fig. 5c). In contrast, CNGRC-yCD/5-FC treatment did not result in cell death for all of the low-APN-expressing tumor cells, which had similar viability levels to the yCD/5-FC and 5-FC treatment group (Fig. 5d).

## Discussion

Targeted tumor therapy is always desired to increase the drug/treatment efficacy and decrease the side effects [27]. APN is a zinc-dependent ectoenzyme that possesses the enzyme activity of removing N-terminal neutral amino acids of proteins and is expressed on the cell membrane of various cell types [28, 29]. The function of APN has been demonstrated in modulating cell migration, invasion, and morphogenesis [6, 28–30]. One recent application employing APN is its effective binding affinity as a receptor with the NGR motif, which has been extensively studied and applied in the development of angiogenic targeting drugs [6, 7, 28, 29]. The advantages of APN as a biomarker for tumor targeting include the following: (1) although APN has been found to express in both normal and angiogenic endothelial cells, it has been suggested that the tumor-targeting property of NGR-drug conjugates only recognizes the APN expressed in angiogenic vessels but not in normal ones due to their different post-translations [31, 32]; (2) it has been demonstrated that APN is overexpressed in a number of tumor cells and plays a crucial modulator role in tumor angiogenesis, metastasis, and survival [16, 28]. Thus, the NGR-drug conjugates could potentially target to APN in both endothelial and tumor cells within the tumor tissues simultaneously and then damage both angiogenesis and tumor growth.

Many new evidences reveal that, in contrast to previously reported antibodies armed with chemo drugs, targeting peptides are potentially other viable candidates to target specific tumor sites owing to their low molecular weights (i.e. making them easier to manipulate) and low immunogenicity, which is particularly important for patients who need prolonged and repeated treatments [7]. It has already been demonstrated that NGR-containing peptides provide the properties of high stability, low immunogenicity, and rapid association with its receptor in vivo [7, 8, 33]. NGR-containing peptides fused with antitumor molecules such as CNGRC-TNF [7] and TF-CNGRC [13] are currently under clinical trials. Our targeting enzyme-prodrug design utilized the NGR motif to deliver the therapeutic enzyme (i.e. the cytosine deaminase) to the tumor site. This study demonstrated that the CNGRC-yCD fusion protein retained APN binding affinity on high-APN-expressing cells and the combined treatment of CNGRC-yCD protein with 5-FC resulted in a significant and selective high-APN-expressing cell death. These promising initial in vitro results, employing the CNGRC-yCD/5-FC system as another candidate for antitumor targeting therapy, would make it feasible to proceed with further in vivo preclinical studies.

The drug 5-FU has been a first-line chemo drug for systemic cancer treatment (e.g. for colorectal, breast, head and neck cancers, and cancers of the aerodigestive tract) [34]. However, clinical evidence has also verified its high cytotoxicity and side effects due to lack of specificity in tumor treatment [18, 19]. Our previous studies and those of others have shown promising evidences about fusing CD with targeting molecules (e.g. EGF) for tumor treatment [17–20, 25]. The results of this study showed that in the CNGRC-yCD/5-FC treatment group, the viabilities of high-APN-expressing human tumor cell lines and endothelial cells were significantly decreased compared to those of low-APN-expressing tumor cell lines. Moreover, the viabilities of high-APN-expressing human tumor cell lines and endothelial cells after treatment with CNGRC-yCD/5-FC correlated well with those treated with 5-FU alone (Fig. 5c), clearly indicating that 5-FU is the major, if not the only, source of cytotoxicity of the CNGRC-yCD/5-FC APN-targeting enzyme-prodrug system. Thus, our current enzyme-prodrug design using NGR to direct the CD/5-FC combination prodrug system could also be a viable antitumor approach to reduce side effects significantly.

Finally, the determined *V*_*max*_ and K_*m*_ values for CNGRC-yCD catalyzed 5-FC transformation to 5-FU reaction were 101.1 ± 5.2 μm/min/μg and 0.56 ± 0.09 mM, respectively, which were similar to those reported previously, i.e. the reported *V*_*max*_ and K_*m*_ values were 20 ~ 80 μm/min/μg and 0.4 ~ 0.8 mM, respectively [20, 22]. These data together with the expression and purification method would provide useful guidance for further preclinical drug-candidate studies.

## Conclusion

We have demonstrated that the CNGRC-yCD fusion protein had significant binding affinities to high-APN-expressing endothelial cells (HUVECs) and various types of human tumor cells, and showed high enzyme activity to convert 5-FC to 5-FU, thus resulting in increased cell death of all high-APN-expressing cells. These promising results encourage further in vivo preclinical studies using the CNGRC-yCD/5-FC combination for future more selective and efficacious targeted antitumor therapy with low systemic side effects.

## Abbreviations

5-FC: 5-fluorocytosine
5-FU: 5-fluorouracil
APN: aminopeptidase N
BSA: bovine serum albumin
CD: cytosine deaminase
cDNA: complementary DNA
DMSO: dimethyl sulfoxide
DNase: deoxyribonuclease
EDTA: ethylenediaminetetraacetic acid
EGF: epidermal growth factor
ELISA: enzyme-linked immunosorbent assay
FACScan: fluorescence activated cell scan
FBS: fetal bovine serum
HRP: horseradish peroxidase
IgG: immunoglobulin G
IPTG: isopropyl β-D-1-thiogalactopyranoside
LB broth: Luria-Bertani broth
MTT: 3-(4,5-dimethylthiazol-2-yl)-2,5-diphenyltetrazolium bromide
NTA: nitrilotriacetic acid
PBS: phosphate buffered saline
PBST: phosphate buffered saline with 0.05 % Tween 20
PCR: polymerase chain reaction
PMSF: phenylmethylsulfonyl fluoride
PSI: pounds per square inch
SDS–PAGE: sodium dodecyl sulfate polyacrylamide gel electrophoresis
TF: tissue factor
TMB: 3,3 ‘,5,5 ‘-tetramethylbenzidine
TNF-α: tumor necrosis factor-α
UV: ultraviolet
UV–VIS: ultraviolet–visible spectroscopy
yCD: yeast cytosine deaminase
